# Supplementary material for: Structural basis for VLDLR recognition by eastern equine encephalitis virus
Source: Nat Commun. 2024 Aug 2;15:6548. doi: 10.1038/s41467-024-50887-9 (PMC11297306; doi:10.1038/s41467-024-50887-9)
Supplement: Supplementary file 1 — Supplementary Information [file 41467_2024_50887_MOESM1_ESM.pdf]

Supplementary information

Structural basis for VLDLR recognition by eastern equine encephalitis virus

Pan Yang[1,#], Wanyu Li[1,#], Xiaoyi Fan[1], Junhua Pan[1,2], Colin J. Mann[1], Haley Varnum[1], Lars E. Clark[1], Sarah A. Clark[1], Adrian Coscia[1], Himanish Basu[3], Katherine Nabel Smith[1], Vesna Brusic[1], Jonathan Abraham[1,4,5,*]

[1]Department of Microbiology, Blavatnik Institute, Harvard Medical School, Boston, MA, USA
[2]Biomedical Research Institute and School of Life and Health Sciences, Hubei University of Technology, Wuhan, Hubei, China
[3]Department of Immunology, Blavatnik Institute, Harvard Medical School, Boston, MA, USA
[4]Department of Medicine, Division of Infectious Diseases, Brigham & Women's Hospital, Boston, MA, USA
[5]Center for Integrated Solutions in Infectious Diseases, Broad Institute of Harvard and MIT, Cambridge, MA, USA

[#]These authors contributed equally.
*Correspondence to: jonathan_abraham@hms.harvard.edu

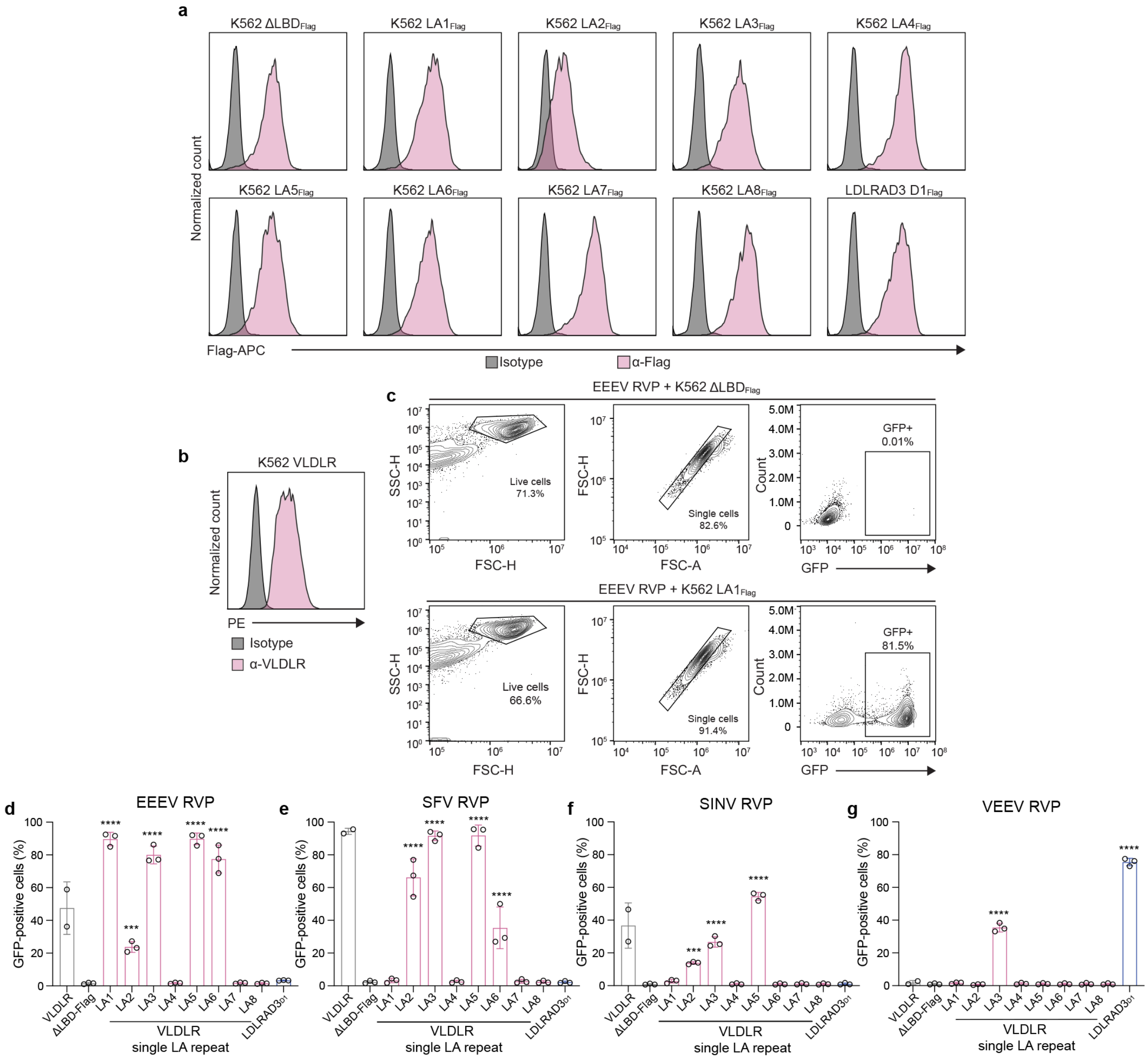

Supplementary Fig. 1. Alphavirus LA repeat dependency mapping studies. a, Anti-flag antibody immunostaining of K562 cell lines stably expressing the indicated constructs measured using flow cytometry. APC: allophycocyanin. **b**, Anti-VLDLR antibody staining of K562 cells stably expressing a full-length VLDLR construct, which does not contain a flag-tag. PE: *R*-phycoerythrin. **c**, Example of flow cytometry gating strategy for quantification of GFP-positive cells after EEEV reporter virus particle (RVP) infection of K562 cells expressing ΔLBD-FLAG (top panels) or a VLDLR LA1 single LA repeat construct (bottom panels). The percentage of cells in each gate is shown. **d**–**g**, K562 cells stably expressing the constructs shown in **a** and **b** were infected with GFP-expressing RVPs for EEEV FL-91-469 (**d**), SFV (**e**), SINV (**f**), or VEEV (**g**) and infection was measured using flow cytomtery. The results of these experiments are summarized in Figure 1b. The flow cytometry gating strategy shown in **c** was used to quantify alphavirus RVP infection for data shown in panels **d**–**g** and Figures 2c, 2f, 3d, and 3e. Data are mean± s.d. from two or three experiments performed in triplicate (**d**–**g**) (n=3 independent experiments, or n=2 independent experiments for full-length VLDLR). One-way ANOVA with Dunnett's multiple comparisons test, compared to ΔLBD-FLAG: EEEV RVP LA2 ***P=0.0002 (**d**); SINV RVP LA2 ***P=0.0008 (**f**); in all other cases, ****P<0.0001. Source data are provided as a Source Data file.

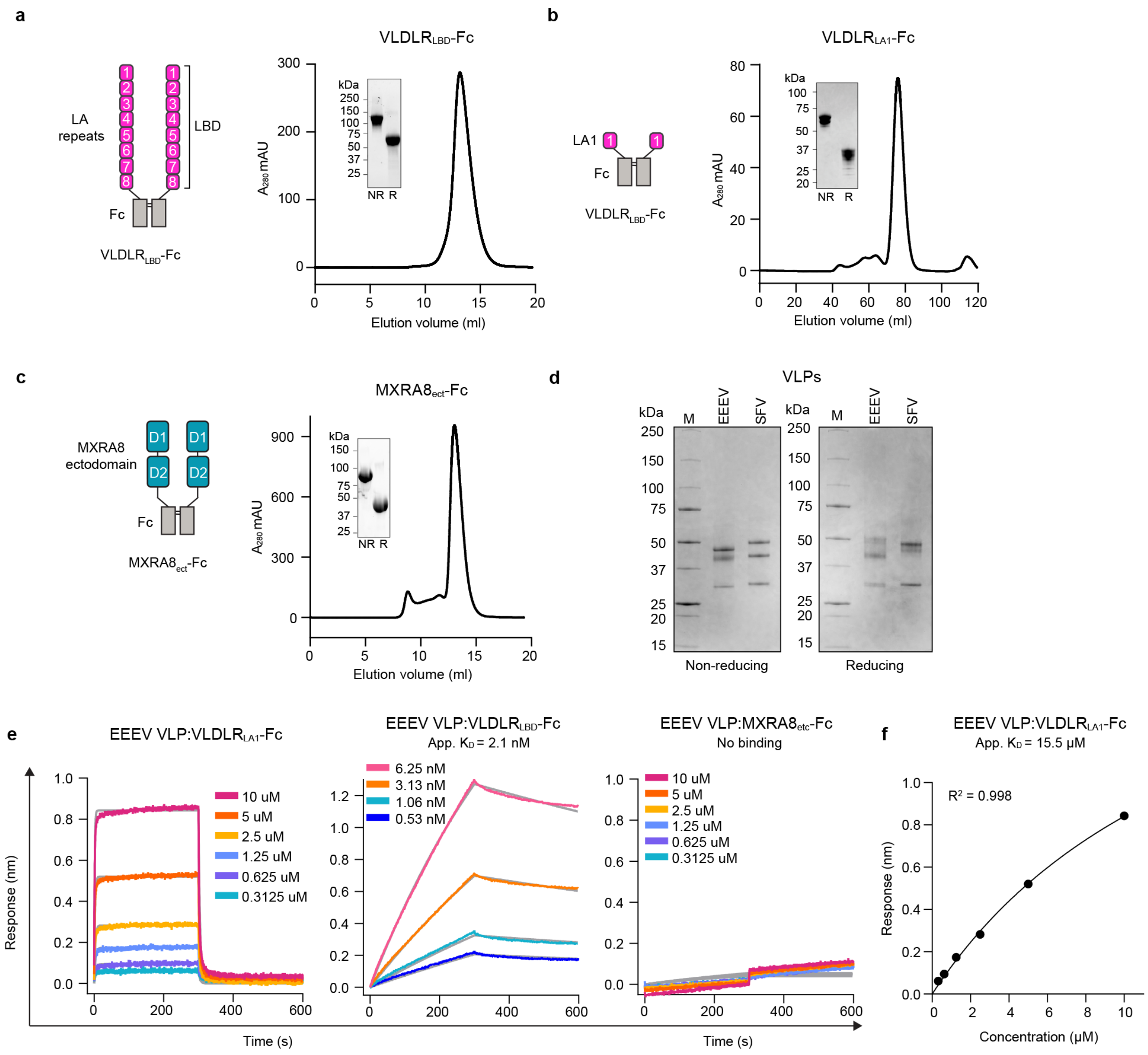

Supplementary Fig. 2. Binding experiments with Fc fusion proteins and virus-like particles. **a**–**c**, Size exclusion chromatography trace of VLDLR$_{LBD}$-Fc (**a**), VLDLR$_{LA1}$-Fc (**b**), and MXRA8$_{ect}$-Fc (**c**). Insets are SDS-PAGE gels of pooled peak fractions. VLDLR$_{LBD}$–Fc and MXRA8$_{ect}$–Fc were visualized using a stain-free imaging system and VLDLR$_{LA1}$-Fc gel is a Coomassie-stained gel. Experiments were performed at least three times and representative traces and gels are shown. NR, nonreducing; R, reducing. A schematic depiction of each Fc fusion protein is provided. **d**, Coomassie-stained SDS-PAGE gel of purified VLPs. The experiment was performed twice and representative gel images are shown. **e**, Biolayer interferometry binding analysis of VLDLR$_{LBD}$-Fc, VLDLR$_{LA1}$-Fc, or MXRA8$_{ect}$-Fc with immobilized EEEV PE6 VLPs. Gray lines represent the fit for a 1:1 binding model. For the EEEV VLP:VLDLR$_{LBD}$-Fc interactions, k_a and k_d were measured as 2.37x10^{5} M^{-1}s^{-1} and 4.89x10^{-4} s^{-1}, respectively. **f**, Scatchard plot for the binding data of VLDLR$_{LA1}$-Fc with EEEV PE6 VLPs shown in **e**. The measured apparent affinity is indicated. Uncropped gels for panels a–d and Source data are provided as a Source Data file.

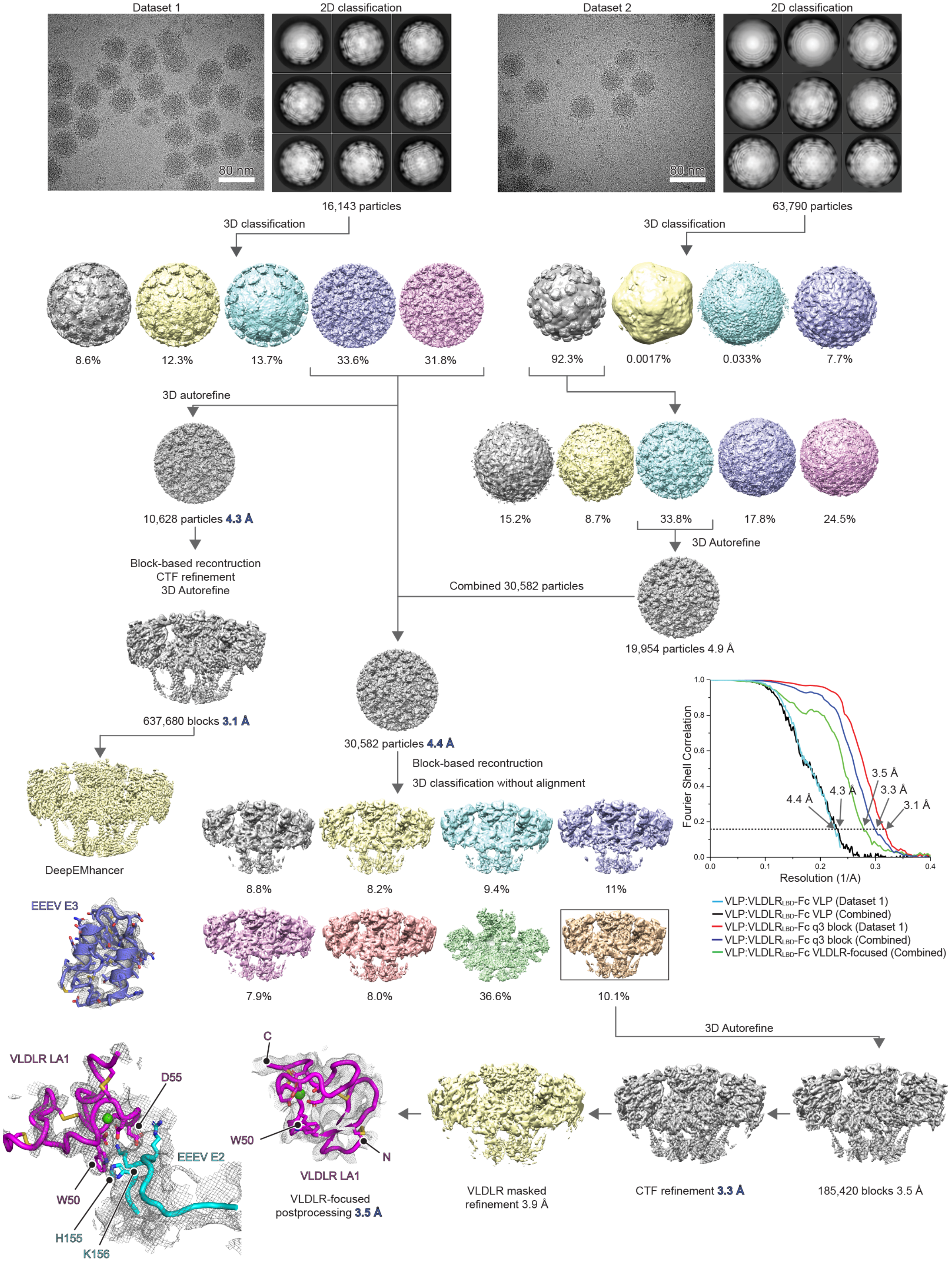

Supplementary Fig. 3. Cryo-EM reconstruction of EEEV virus-like particles bound to $VLDLR_{LBD}$-Fc. Workflow used for cryo-EM data processing of EEEV VLPs bound to $VLDLR_{LBD}$-Fc. Representative micrographs for the first and second datasets are shown. Fourier shell

correlation curves are also shown. The threshold used to estimate the resolution is 0.143. Zoom-in view of cryo-EM density for LA repeat-spike protein contact site is shown (lower left). Maps colored to local resolution and representations of angular distribution or particles are provided in Supplementary Fig. 12a, b, f, and g. See Methods for additional details.

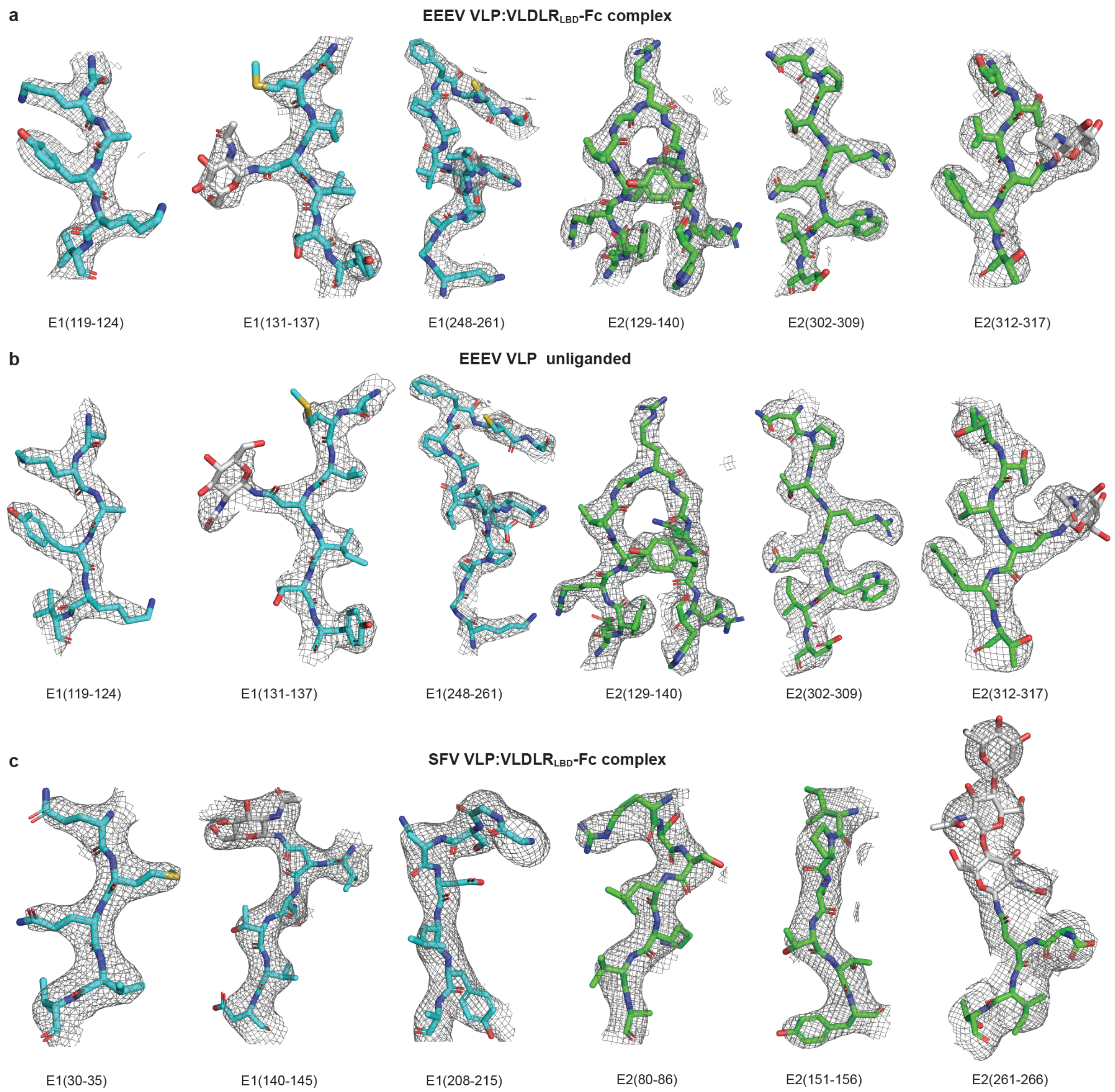

Supplementary Fig. 4. Density for E2 and E1 glycoprotein regions in cryo-EM maps. a–**c**, Density maps are shown overlayed onto stick representation of the indicated polypeptide segments and N-linked glycans for the EEEV VLP:VLDLR$_{LBD}$-Fc complex (**a**), the unliganded EEEV VLPs (**b**), and the SFV VLP:VLDLR$_{LBD}$-Fc complex (**c**).

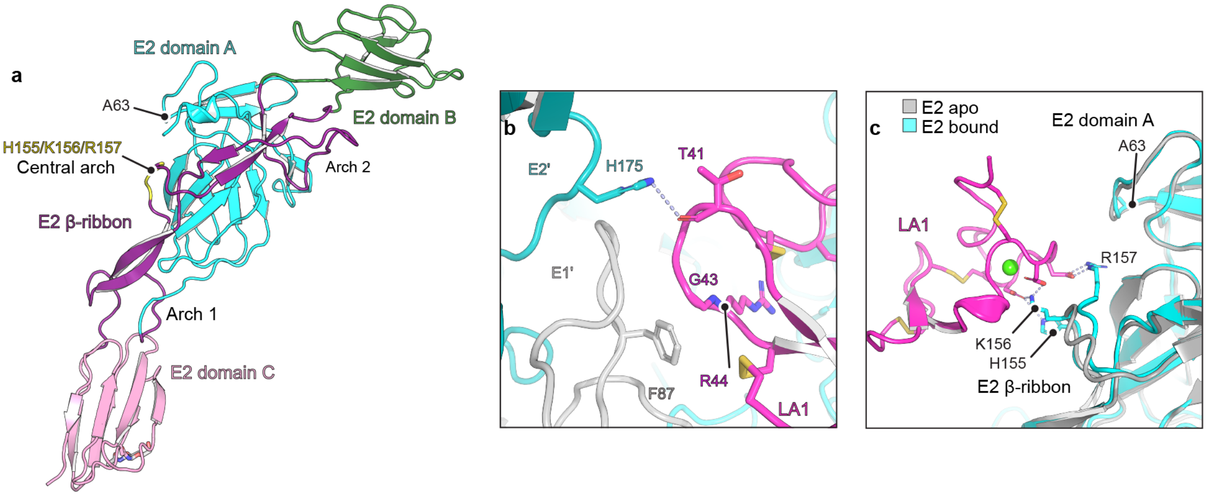

Supplementary Fig. 5. LA repeat binding involves the E2 β-ribbon connector and minimal contacts with the adjacent E2–E1 protomer. a, Ribbon diagram of EEEV E2 determined as part of the complex with VLDLR$_{LBD}$-Fc. E2 residues H155, K156 and R157 in the central arch of the β-ribbon connector make important contacts with VLDLR. A63, in E2 domain A, is the position analogous to VEEV E2 R64, a basic residue that makes important contacts with LDLRAD3[1]. Domain organization is shown as originally described by Voss et al.[2]. **b**, Zoom in view of the LA repeat interactions with the adjacent EEEV spike protein protomer (E2'–E1'). E2' H175 interacts with the backbone carbonyl of LA1 T41, and E1' fusion loop residue F87 makes hydrophobic contacts with the Cα atoms of LA1 G43 and R44. **c**, Zoom in view showing the small conformational changes that occur in the central arch of the EEEV E2 β-ribbon connector loop upon VLDLR binding when the structure of the receptor-bound and unliganded ("apo") EEEV VLPs are compared.

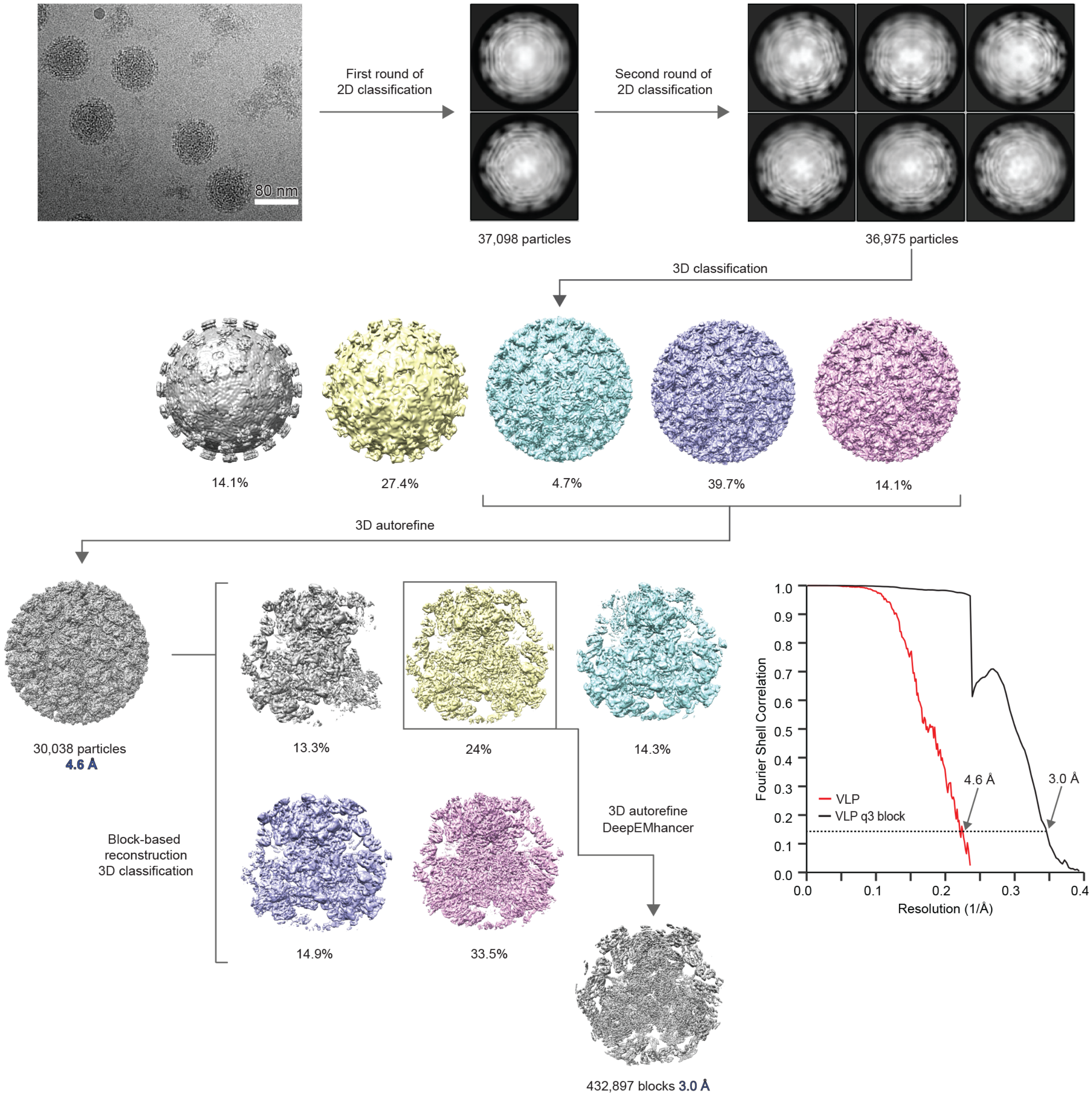

Supplementary Fig. 6. Cryo-EM reconstruction of unliganded EEEV virus-like particles. Workflow used for cryo-EM data processing of unliganded EEEV PE6 VLPs. A representative micrograph is shown. Fourier shell correlation curves are shown. The threshold used to estimate the resolution is 0.143. Maps colored to local resolution and representations of angular distribution or particles are provided in Supplementary Fig. 12c and h. See Methods for additional details.

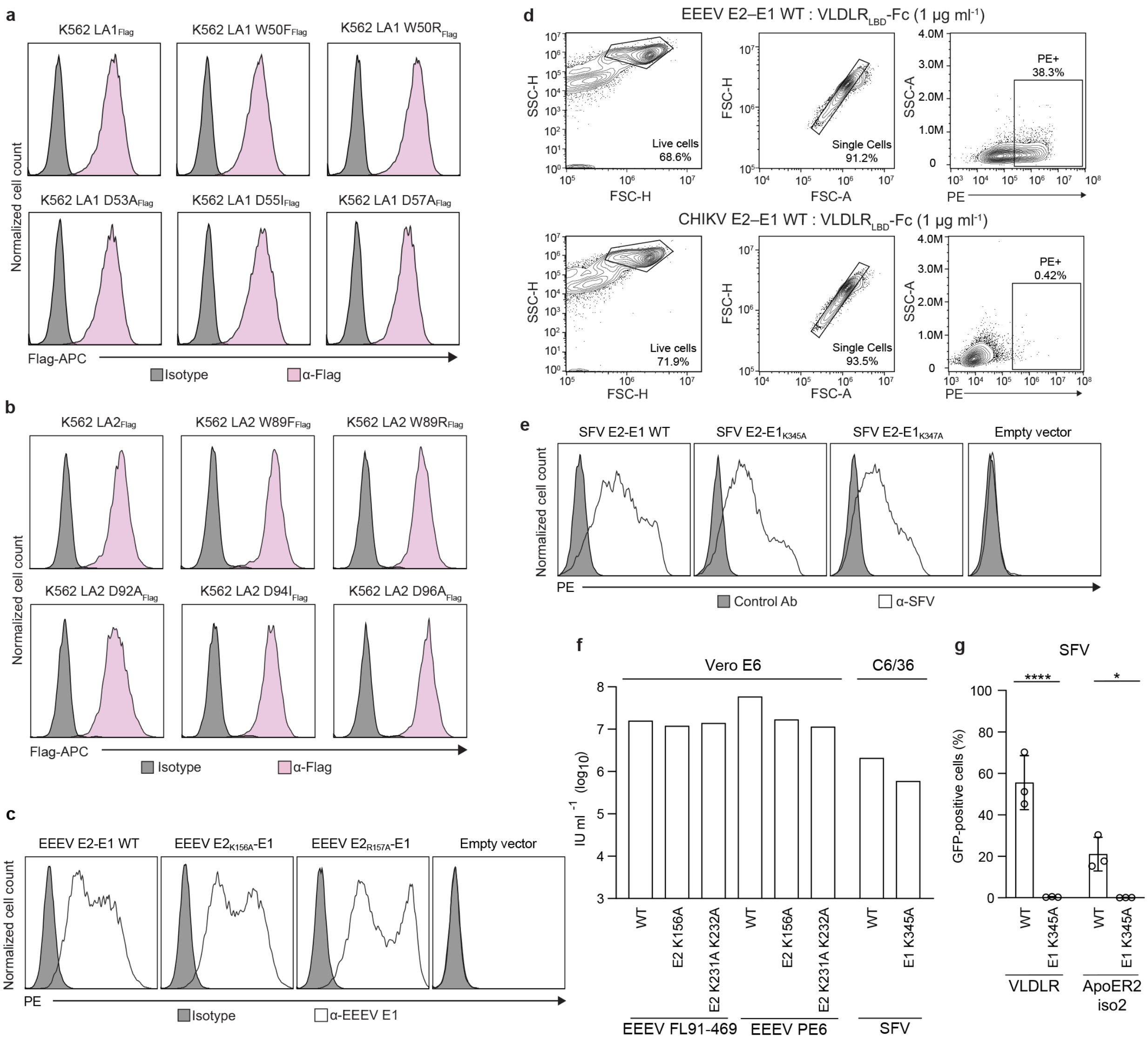

Supplementary Fig. 7. Cell surface expression of constructs used for functional validation of EEEV- or SFV-VLDLR LA repeat contact residues. **a**, Cell surface immunostaining of Flag-tagged constructs used in Figure 2c by allophycocyanin (APC)-conjugated anti-Flag antibody monitored by flow cytometry. **b**, Cell surface immunostaining of Flag-tagged constructs used in Figure 2f by APC-conjugated anti-Flag antibody, as monitored by flow cytometry. **c**, Cell surface immunostaining of HEK293T cells transfected with wild-type (WT) or mutant EEEV FL91-469 spike proteins (E3–E2–[6K/TF]–E1) using anti-EEEV E1 monoclonal antibody. **d**, Example of flow cytometry gating strategy used to quantify Fc fusion protein binding to cells transfected with EEEV or CHIKV spike proteins for data shown in Figures 2d and 2g. PE: *R*-phycoerythrin. **e**, Cell surface immunostaining of HEK293T cells transfected with wild-type or mutant SFV spike proteins using mouse anti-SFV ascitic fluid. In **c** and **e**, staining was detected by PE-conjugated goat anti-mouse IgG $F(ab')_2$ fragment and monitored by flow cytometry. **f**, Representative RVP titers on Vero E6 cells or C6/36 (*Aedes albopictus*) cells. Titers are calculated as infectious unit per milliliter (IU ml^{-1}) as determined by a limiting dilution method in cells 24 h post-infection. See methods for additional details. **g**, K562 cells expressing human VLDLR or ApoER2 iso2 were infected with GFP-expressing wild-type SFV RVPs or RVPs bearing the E1 K345A mutation. Infection was

monitored by flow cytometry. Data are mean ± s.d. from three experiments performed in triplicates (n=3 independent experiments). Two-way ANOVA with Dunnett's multiple comparisons test, ****P<0.0001, *P=0.02. Source data are provided as a Source Data file.

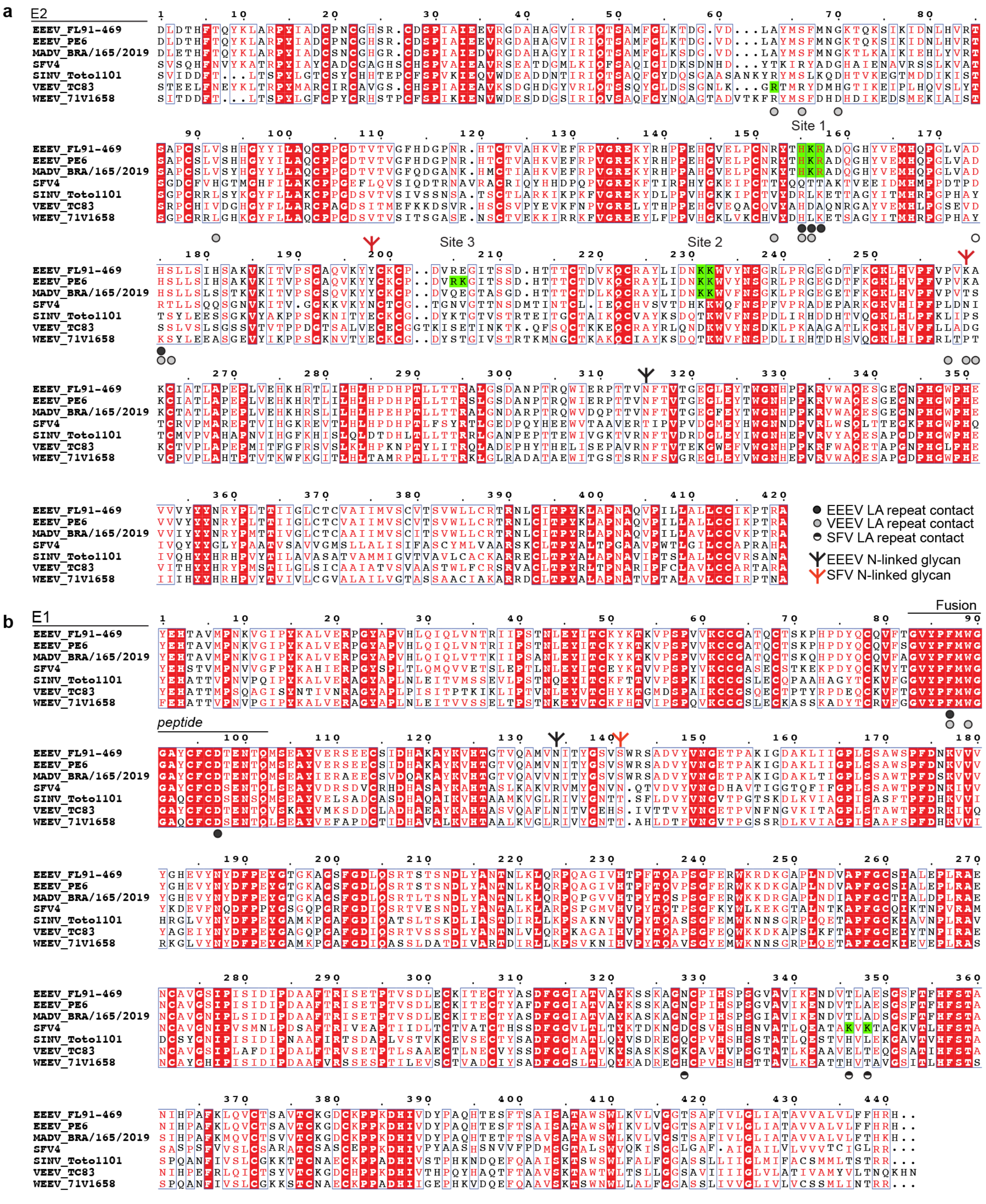

Supplementary Fig. 8. Sequence alignment of alphavirus E2 and E1 glycoproteins. Sequence alignment of the E2 (**a**) and E1 (**b**) glycoproteins of eastern equine encephalitis virus (EEEV), western equine encephalitis virus (WEEV), Venezuelan equine encephalitis virus (VEEV), Madariaga virus (MADV), Semliki Forest virus (SFV), and Sindbis virus (SINV). E2 and E1 residues that are within 4 Å of the LDLR class A (LA) repeat in structures are shown. VEEV contact residues are based on the VEEV VLP:LDLRAD3$_{D1}$ structure (PDB ID 7FFF)[1]. EEEV and

SFV N-linked glycan sites are also indicated. Red background highlights residues completely conserved in all sequences aligned. Boxed residues highlight positions where a single majority residue or multiple chemically similar residues could be identified. Such residues are highlighted in red. The key E2 and E1 basic residues that contact the LA repeat Ca^{2+}-coordinating acidic residues are highlighted in green. Additional basic residues involved in contacting LA repeats described in separate studies (sites 2 and 3) are also shown[3,4]. The strain information and accession numbers are as follows: EEEV Florida (FL) 91-469 (GenBank: Q4QXJ7.1), EEEV PE6 (GenBank: AAU95735.1), MADV BRA/165/2019 (GenBank: UDP68813.1), SFV 4 (GenBank: AKC01668.1), SINV Toto1101 (AKZ17594.1), VEEV TC-83 (GenBank: AAB02517.1), and WEEV 71V1658 (GenBank: NP_640331.1). The panels were generated using ESPript 3.0[5].

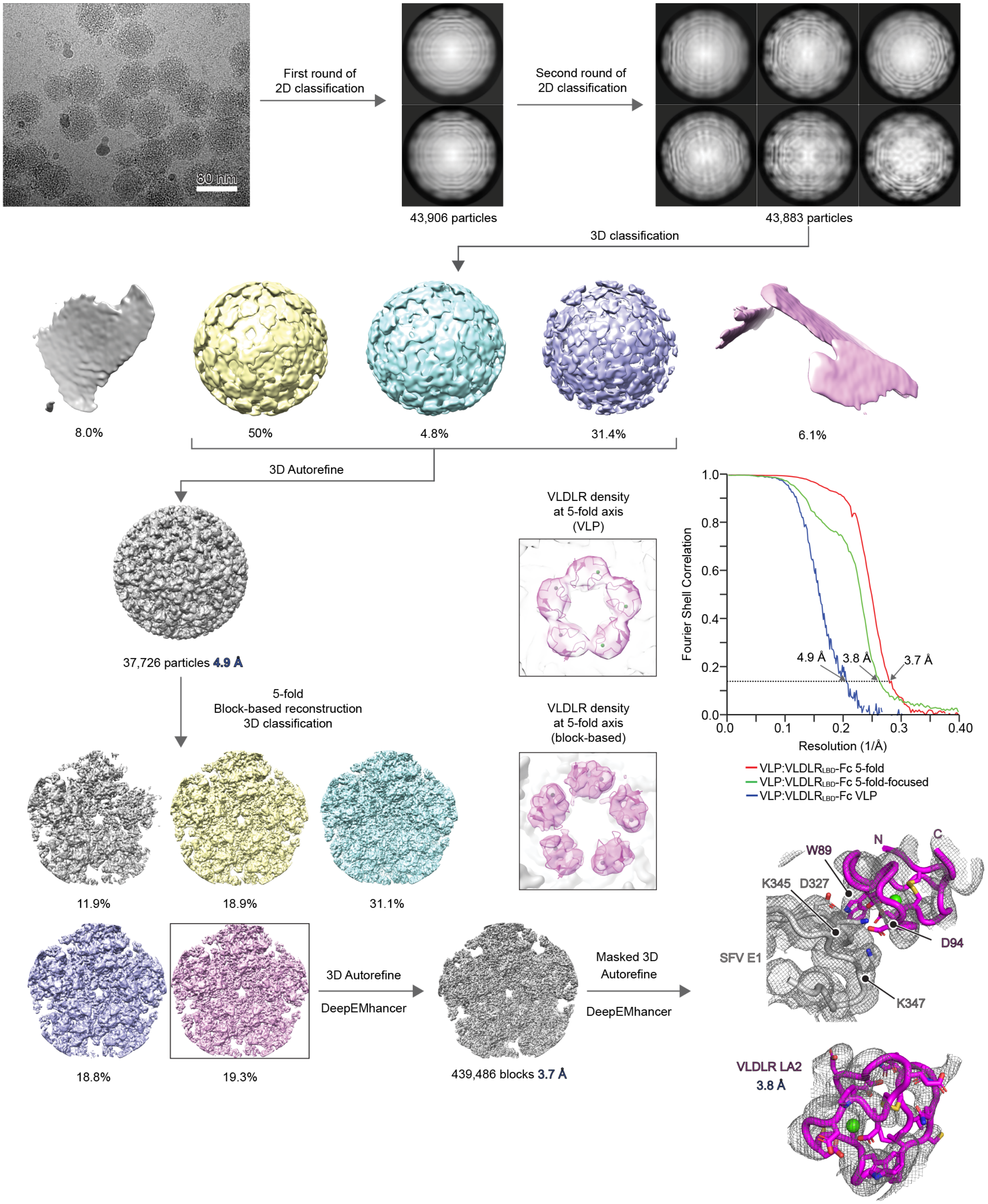

Supplementary Fig. 9. Cryo-EM reconstruction of SFV virus-like particles bound to VLDLR$_{LBD}$-Fc. Workflow used for cryo-EM data processing and block-based reconstructions of SFV VLPs bound to VLDLR$_{LBD}$-Fc. A representative micrograph is shown. Fourier shell correlation curves are also shown. The threshold used to estimate the resolution is 0.143. A zoom in view of cryo-EM density for LA repeat-spike protein contact site is shown (lower right). Maps colored to

local resolution and representations of angular distribution or particles are provided in Supplementary Fig. 12d, e, i, and j. See methods section for additional information.

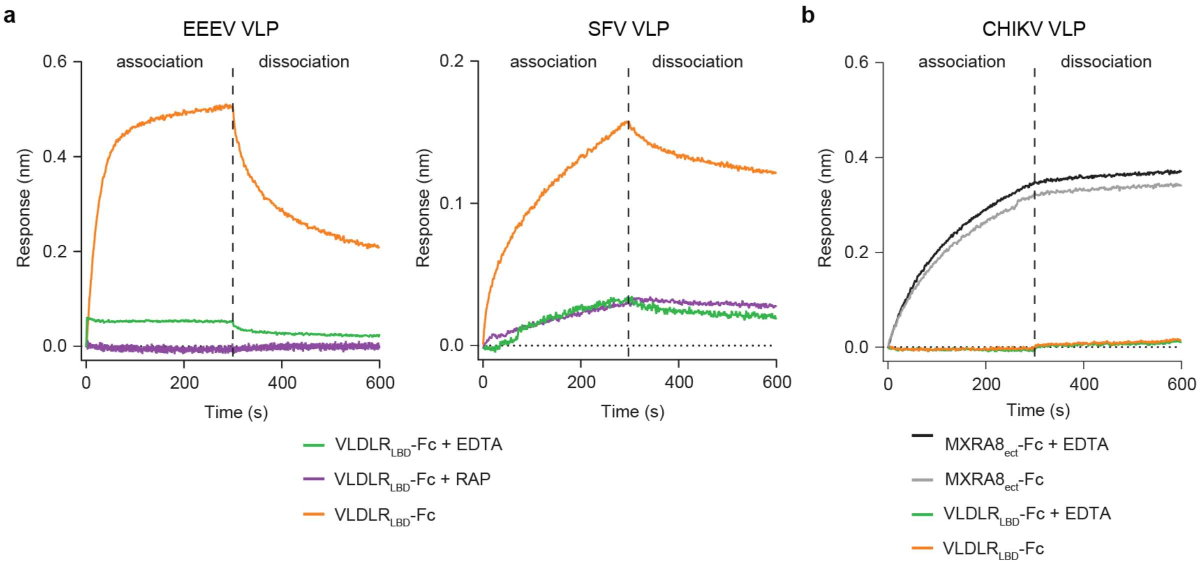

Supplementary Fig. 10. EDTA treatment disrupts EEEV and SFV binding to VLDLR. Biolayer interferometry binding analysis of 20 nM $VLDLR_{LBD}$-Fc with immobilized EEEV or SFV VLPs (**a**), in the presence or absence of 10 mM EDTA or 100 μg ml^{-1} (2.6 μM) RAP included in the association phase. Immobilized CHIKV VLPs and $MXRA8_{ect}$-Fc were used as controls (**b**). Experiments were independently performed twice, and representative sensorgrams are shown. Source data are provided as a Source Data file.

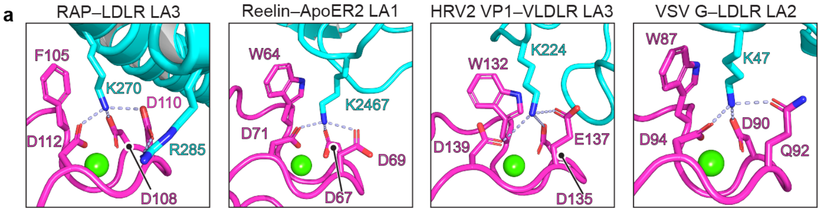

b

Complex	Burried surface area (Å²)	PDB ID
SFV E2–E1 VLDLR LA2	238	8UA8
SFV E2–E1 VLDLR LA3	402	8IHP
EEEV E2–E1 VLDLR LA1	645	8UA4
VEEV E2–E1 LDLRAD3	1,111	7FFF
CHIKV E2–E1 MXRA8	2,212	6JO8
VSV G LDLR LA3	791	5OY9
VSV G LDLR LA2	858	5OYL
RAP LDLR LA3	404	2FCW
RAP LDLR LA4	388	2FCW

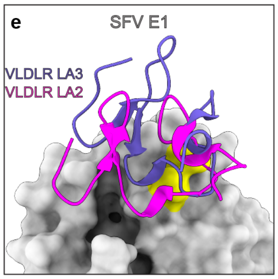

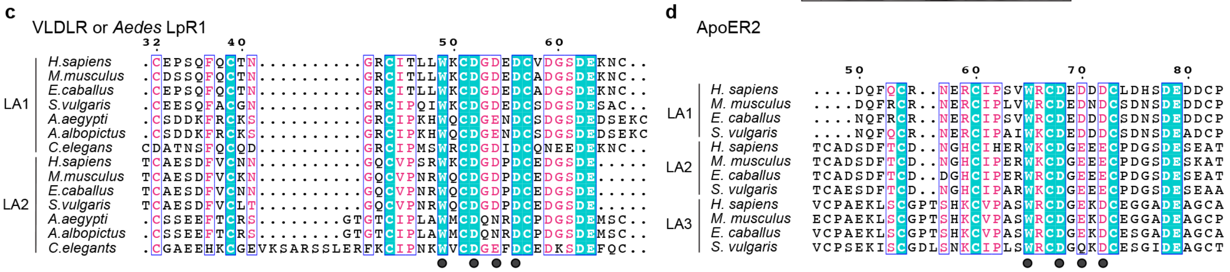

Supplementary Fig. 11. Comparison with other LA repeat-bound structures, buried surface area calculations, and LA repeat sequence alignments. a, Zoom in view of contacts RAP (PDB ID: 2FCW)[6], Reelin (PDB ID: 3A7Q)[7], human rhinovirus 2 (HRV2) VP1 (PDB ID: 3DPR)[8], and vesicular stomatitis virus (VSV) glycoprotein (G) (PDB ID: 5OYL)[9] make with the Ca^{2+}-coordinating acidic residues and central aromatic residue in LA repeats. LA repeats are shown in magenta; viral glycoproteins and ligands are shown in cyan. **b**, Buried surface area calculations for the indicated complexes. **c**, Alignment of VLDLR and lipophorin receptor 1 (LpR1) LA1 and LA2 amino acid sequences. GenBank accession numbers are *H. sapiens* VLDLR (GenBank NP_003374.3), *M. musculus* VLDLR (AAH13622.1), *E. caballus* VLDLR (GenBank XP_023483037), *S. vulgaris* VLDLR (GenBank XM_014880599.1), *A. aegypti* LpR1 (GenBank JN411069.1), *A. albopictus* LpR1 (GenBank JAC13440), and *C. elegans* VLDLR (GenBank NM_182223.6). **d**, Alignment of ApoER2 LA1, LA2, and LA3 amino acid sequences. GenBank accession numbers are *H. sapiens ApoER2* isoform 2 (GenBank NM_004631.5), *M. musculus* ApoER2 (GenBank XP_036019651), *E. caballus* ApoER2 (GenBank XP_023485552), and *S. vulgaris* ApoER2 (GenBank XM_014870608.1). In **c** and **d**, cyan background highlights residues completely conserved in all aligned LA repeats. Boxed residues highlight positions where a single majority residue or multiple chemically similar residues could be identified. Such residues are highlighted in magenta. The alignment representations were generated using ESprit3[5]. Black circles indicate residues that contact alphavirus spike proteins. **e**, Structures of SFV bound to $VLDLR_{LBD}$-Fc reported here and the structure of SFV bound to $VLDLR_{LA3}$-Fc recently reported by Cao et al[10]. E1 is shown in surface representation, and LA repeats are shown in ribbon diagram. E1 K345 and K347 are shown in yellow.

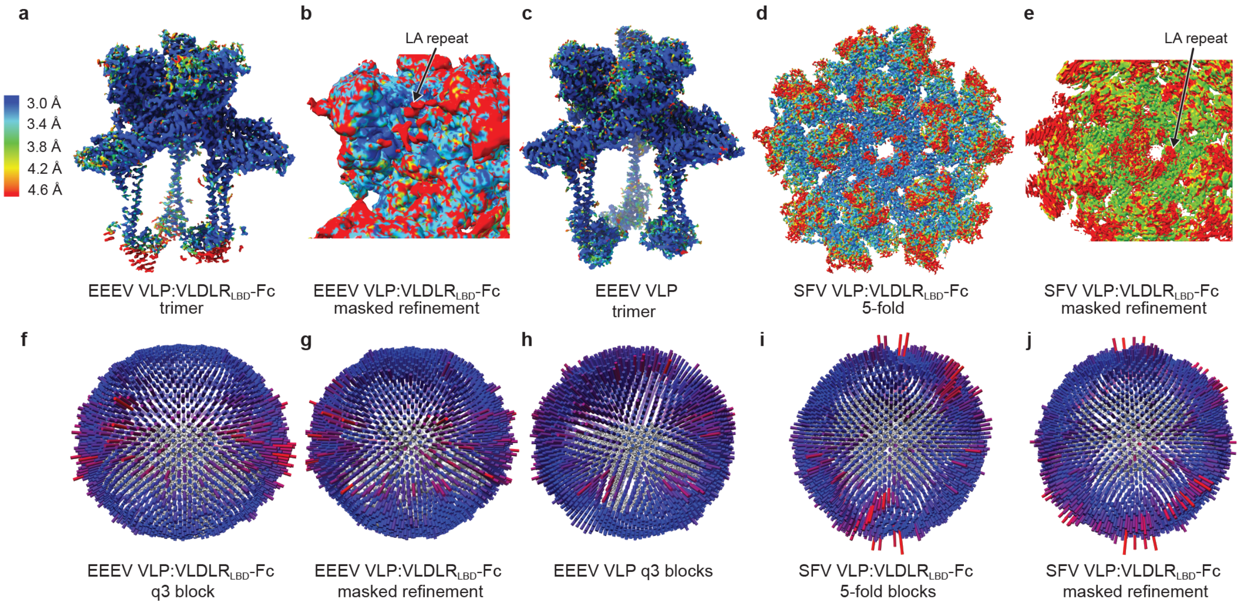

Supplementary Fig. 12. Map local resolution estimates and representations of particle angular distributions. a–e, Estimates of local resolution for the indicated cryo-EM maps generated using ResMap[11]; the scale shown in **a** applies to all panels. For panels **b** and **e**, the LA repeat density indicated by the arrow was the focus of masked refinement. **f–j**, 3D representation of angular distribution of particles generated using Relion 3.1[12].

Supplementary Table 1. Cryo-EM data collection and validation statistics.

	EEEV VLP:VLDLR$_{LBD}$-Fc		SFV VLP:VLDLR$_{LBD}$-Fc	EEEV VLP
	Dataset 1	Dataset 2		
Magnification	81,000		105,000	81,000
Voltage (kV)	300		300	300
Pixel Size (Å)	1.06		0.825	1.06
Electron dose (e/Å^2)	53	54.1	54.9	54.1
Defocus range (μm)	-1.0 to -2.5		-1.5 to -2.5	-1.0 to -2.5
VLP reconstruction				
Initial particles	16,143	63,790	43,906	37,098
Final particles	10,628	19,954	37,726	30,038
Symmetry imposed			Icosahedral symmetry	
FSC threshold	0.143		0.143	0.143
Map resolution (Å)	4.3	4.4	4.9	4.6
Block-based reconstruction				
	Dataset 1	Combined		
Initial Blocks	637,680	1,834,920	2,263,560	1,802,280
Final Blocks	637,680	185,420	439,486	432,897
Symmetry imposed	C1		C1	C1
FSC threshold	0.143		0.143	0.143
Map resolution (Å)	3.1	3.3	3.7	3.0
**Model refinement and validation**				
Initial model used			AlphaFold2	
R.m.s deviations				
Bonds lengths (Å)	0.65		0.65	0.70
Bonds angles (°)	0.77		0.79	0.76
Validation				
Clashscore	7		8	7
Favored (%)	99		99	99
Allowed (%)	1		0	1
Disallowed (%)	0		0	0

Supplementary References

1 Ma, B., Huang, C., Ma, J., Xiang, Y. & Zhang, X. Structure of Venezuelan equine encephalitis virus with its receptor LDLRAD3. *Nature* **598**, 677-681 (2021). https://doi.org:10.1038/s41586-021-03909-1

2 Voss, J. E. *et al.* Glycoprotein organization of Chikungunya virus particles revealed by X-ray crystallography. *Nature* **468**, 709-712 (2010). https://doi.org:10.1038/nature09555

3 Adams, L. J. *et al.* Structural and functional basis of VLDLR usage by Eastern equine encephalitis virus. *Cell* **187**, 360-374 e319 (2024). https://doi.org:10.1016/j.cell.2023.11.031

4 Duanfang Cao, B. M., Ziyi Cao, Xiaoyu Xu, Xinzheng Zhang, Ye Xiang. The receptor VLDLR binds East Equine Encephalitis virus through multiple distinct modes. *bioRxiv* (2023). https://doi.org:https://doi.org/10.1101/2023.11.30.569340

5 Robert, X. & Gouet, P. Deciphering key features in protein structures with the new ENDscript server. *Nucleic Acids Res* **42**, W320-324 (2014). https://doi.org:10.1093/nar/gku316

6 Fisher, C., Beglova, N. & Blacklow, S. C. Structure of an LDLR-RAP complex reveals a general mode for ligand recognition by lipoprotein receptors. *Mol Cell* **22**, 277-283 (2006). https://doi.org:10.1016/j.molcel.2006.02.021

7 Yasui, N., Nogi, T. & Takagi, J. Structural basis for specific recognition of reelin by its receptors. *Structure* **18**, 320-331 (2010). https://doi.org:10.1016/j.str.2010.01.010

8 Querol-Audi, J. *et al.* Minor group human rhinovirus-receptor interactions: geometry of multimodular attachment and basis of recognition. *FEBS Lett* **583**, 235-240 (2009). https://doi.org:10.1016/j.febslet.2008.12.014

9 Nikolic, J. *et al.* Structural basis for the recognition of LDL-receptor family members by VSV glycoprotein. *Nat Commun* **9**, 1029 (2018). https://doi.org:10.1038/s41467-018-03432-4

10 Cao, D., Ma, B., Cao, Z., Zhang, X. & Xiang, Y. Structure of Semliki Forest virus in complex with its receptor VLDLR. *Cell* **186**, 2208-2218 e2215 (2023). https://doi.org:10.1016/j.cell.2023.03.032

11 Kucukelbir, A., Sigworth, F. J. & Tagare, H. D. Quantifying the local resolution of cryo-EM density maps. *Nat Methods* **11**, 63-65 (2014). https://doi.org:10.1038/nmeth.2727

12 Zivanov, J. *et al.* New tools for automated high-resolution cryo-EM structure determination in RELION-3. *Elife* **7** (2018). https://doi.org:10.7554/eLife.42166
